# Linking Leeds: A Social Prescribing Service for Children and Young People

**DOI:** 10.3390/ijerph19031426

**Published:** 2022-01-27

**Authors:** Melissa Brettell, Clare Fenton, Ethan Foster

**Affiliations:** 1 Leeds and York Partnership NHS Foundation Trust, Leeds LS15 8ZB, UK; 2 COMIC (Child Oriented Mental health Intervention Centre) Research, University of York, York YO10 5DD, UK; clarefenton@nhs.net; 3 Linking Leeds, Leeds LS7 3EX, UK; ethan.foster@commlinks.co.uk

**Keywords:** social prescribing, mental health, children and young people, CAMHS

## Abstract

The use of social prescribing interventions for common mental health issues is expanding as clinicians seek to diverge from the traditional medical model of treatment. This intervention allows for the referral of patients to a nonclinical social activity via a link worker. Evidence for the benefits of social prescribing is growing. Most evidence is based on adults; however, a smaller number of studies involving children and young people have produced encouraging results. This evaluation reports on data routinely collected by the Linking Leeds service between 9 January 2019–11 January 2020. Linking Leeds provides Social Prescribing for people aged 16 years and above; however, the current paper focuses on service users aged between 16 and 25. Their aim is to connect people to services and activities in their community in order to benefit overall health and mental wellbeing. This evaluation of the Linking Leeds program supports the growing body of evidence to support the benefits social prescribing can have on young people’s mental health. Two main mechanisms were identified which underpin social prescribing in young people: social connectedness and behavioural activation.

## 1. Introduction

### 1.1. Mental Health Services for Young People

Demand for mental health services in children is increasing, with one in eight 5–19-year-olds suffering from a mental illness [1]. Self-harm is more common among young people than any other age group [2] and globally, depression is now one of the leading causes of illness and disability among adolescents [3]. Suicide rates in adolescents have also increased; between 2010 and 2017, one study found an increase of 7.9% per year [4]. 

Young people who experience mental health disorders are more likely than their peers to engage in risk behaviours. A 2017 survey found that 11–16-year-olds with a mental disorder were more likely to have self-harmed or attempted suicide at some point (25.5%) than those without a disorder (3.0%). They were also more likely to engage in tobacco, alcohol and illicit drug use, in addition to being at higher risk of exclusion from school [1].

Half of all mental illnesses begin by age 14 and three-quarters begin by age 24 [5]. According to the World Health Organisation, the consequences of not addressing adolescent mental health conditions extend to adulthood and have a detrimental effect on both physical and mental health [3]. Currently, in the UK, there are long waiting times for treatment from Child and Adolescent Mental Health Services (CAMHS), with one in five children with a mental disorder waiting over six months for contact with a mental health specialist [1]. In 2016, the Royal College of Nurses carried out a survey involving 631 CAMHS nurses to gain insight into their views regarding the service. Common issues arose which pertained to delays in service users accessing treatment. These include delays in patients getting appointments, the inability to give patients as many appointments or as much care as they need, high thresholds or qualifying criteria to get care and many referrals not actually receiving NHS care [6]. In 2018, Young Minds found that 76% of parents and carers thought their child’s mental health had deteriorated while waiting to access CAMHS [7].

In the UK, talking therapies are widely used to treat children and young people with mental health difficulties [8]. Although they have a strong evidence base, drop-out rates are high—between 28% and 75% [9]. The Department of Health suggests the need for more innovative interventions and increased third sector involvement [10].

### 1.2. Social Prescribing

One such innovative approach that is growing in use is social prescribing. Social prescribing is defined as ‘enabling healthcare professionals to refer patients to a link worker…to co-design a nonclinical social (activity) to improve their health and well-being’ [11]. Other terms broadly equivalent to social prescribing include ‘community referral’, ‘community links’, and ‘arts on prescription’ [12]. Schemes delivering social prescribing can involve a range of activities that are typically provided by voluntary and community sector organisations. Examples include volunteering, arts activities, group learning, gardening, befriending, cookery and a range of sports [13].

Organisations differ in their skillset and staff can include both voluntary and paid workers. Staff backgrounds and knowledge can vary from qualifications in counselling and family therapy and in-depth knowledge of support available to service users, including welfare benefits and housing, to past experience working in social work or teaching. Others may be qualified for the role due to personal experiences of mental health issues. Services also provide training to staff to ensure they are well equipped to support the service users they will be working with [14,15]. Organisations generally focus on providing support for individuals with mental health and/or psychosocial needs and this support tends to be fairly holistic, depending on the independent needs of service users. For example, some individuals may benefit from practical support with finances and job seeking, whereas others require input more geared towards reducing social isolation. The shared goal of all social prescribing services is to improve wellbeing [14].

In recent years, the NHS has utilised social prescribing as a treatment for common mental health issues as it is designed to support people with a wide range of social, emotional or practical needs. Social prescribing is a key component of Universal Personalised Care, the aim of which is to give service users the same choice and control over their mental and physical health as they would expect in other aspects of their lives. It was also included in the NHS Long Term Plan; NHS England committed to building the infrastructure for social prescribing in primary care with the aim that at least 900,000 people will be referred to social prescribing by 2023–24 [16].

Social prescribing link workers are becoming an integral part of the multi-disciplinary teams in primary care networks (PCNs). The NHS states that this is the biggest investment in social prescribing by any national health system and legitimises community-based activities and support alongside medical treatment as part of personalised care [14].

The evidence base for social prescribing is growing; however, most studies to date have focused on adults. A recent systematic review found that adults engaging in social prescribing interventions reported an increase in confidence [17]. The authors report this was mainly a result of a reduction in social isolation, as the intervention motivated participants to join social groups and build a social network of support. Furthermore, the evidence suggested that applying a co-productive approach to social prescribing gave individuals a sense of control. This enabled their self-confidence to improve in addition to their mood. Similarly, Chatterjee and colleagues examined the outcomes reported from 86 UK social prescribing schemes for adults. Results suggested increases in self-esteem and confidence, improvement in mental well-being and positive mood and reductions in anxiety and depression [18].

Polley et al. conducted a systematic review of fifteen social prescribing programmes. All programmes that utilised validated outcome measures reported some improvement in health and wellbeing; however, the authors highlight that common design issues included a lack of comparative controls and short follow-up durations (six months). In addition to wellbeing measures, the review looked at other factors which should be considered when evaluating social prescribing services. On average, studies reported a 28% reduction in demand for GP services following referral as well as an average of 24% fall in attendance at Accident and Emergency [19]. Researchers attributed these reductions to connecting service users with more appropriate, non-clinical services which better addressed their needs. The majority of these services were community based and included sporting groups, art groups, volunteering and specialist support for issues such as employment. One study also found that engagement with a social prescribing service reduced the use of psychotropic medication, which is costly to health services [20].

Research studies that have focused on specific social prescribing activities have also elicited encouraging results. Hacking et al. surveyed individuals attending 22 different group art projects in England. Self-reported measures of wellbeing, including the Clinical Outcomes in Routine Evaluation (CORE) measure, were taken on first entry to the project during January to March of 2006 and repeated six months later. The questionnaires included three measures: empowerment, mental health and social inclusion. Results showed significant improvements on all three measures [21]. This study is advantageous as it provides details pertaining to participant diagnoses: of those who provided details, 18 had a diagnosis of depression, 4 had schizophrenia, 10 suffered from bipolar disorder, 17 had anxiety with depression and 10 categorised their issues as ‘other’. The authors report the latter category includes individuals suffering from a personality disorder and obsessive/stress-related disorders. Furthermore, the researchers explored whether symptom severity was a predictor of outcomes. The analysis showed that those with more severe mental health problems (i.e., those with clinically significant first-entry CORE scores) particularly benefited from the program in terms of feeling empowered. Empowerment scores for this sub-group increased by 19%, whereas scores for those with less severe difficulties did not increase. Outcomes were not predicted by category of mental health difficulty or adherence to the intervention. From the 61 people who answered the question, the majority (48 people) were still regularly attending their project six months after they filled in their first questionnaire on entry to the project; 44 were attending weekly or more, 3 fortnightly and 1 person attended ‘a few times’. Of 13 people who indicated they had stopped attending, 10 had attended for longer than 3 months and the 3 remaining had attended for a shorter duration but “very frequently”. There was a great variation in the design of projects; however, most projects limited participation to weekly sessions of 1–3 h.

Margrove and colleagues provide additional support for the use of art in social prescribing services. This study is particularly advantageous compared to similar studies due to the use of a control group. Individuals engaging in a 12-week group art course were allocated to one of two groups: an immediate place on the course or a place on the waiting list. At the end of the 12 weeks, those in the intervention group displayed a significant increase in mental wellbeing scores compared to before engaging in the programme. No significant difference across time was found for the control group. Of the intervention group, 96% reported enjoying the course and most of those providing feedback reported gains in confidence (81%) and motivation (88%) [22]. However, limited details are provided regarding the mental health difficulties of participants, with the authors reporting that the service is open to anybody struggling with their mental health. It is unclear whether service users were engaged with other mental health services or the severity of their difficulties.

Clatworthy et al carried out a meta-analysis of gardening-based interventions for adults experiencing mental health difficulties [23]. Of the studies reviewed, four focused on individuals with depression, one studied those with schizophrenia and the remaining five report either ‘mixed’ diagnoses or do not provide any details. It is unclear whether participants were required to have official diagnoses or if difficulties were based on self-report. All reported positive effects of gardening as a mental health intervention for service users, including reduced symptoms of depression and anxiety. There is also support for the effectiveness of group physical exercise. Results from a cross-sectional study analysing data from over 1 million adults in the USA found that although all types of physical exercise were associated with a lower mental health burden, the largest associations were seen for team sports [24].

A number of studies have taken a closer look at specific social prescribing services and their effectiveness. Grant et al. conducted a randomised controlled trial of a social prescribing service in Bristol called the Amalthea Project. This is a liaison organisation that facilitates contact between voluntary organisations and patients in primary care. Eligible patients are those aged 16 years or over with psychosocial problems who general practitioners thought might benefit from contact with the voluntary sector. Common referral reasons include depression, anxiety or stress, substance misuse, social isolation and bereavement or loss. Patients were compared to those receiving routine general practitioner care. The Amalthea group showed significantly greater improvements in anxiety, ability to carry out everyday activities, quality of life and feelings about general health [25]. 

Another service that has elicited positive outcomes is New Routes, a social prescribing service based in the Keynsham, Bath and North-East Somerset area. A detailed pilot evaluation was conducted, and the study presents an analysis of all service users referred to New Routes over a three-year period from October 2009 through to September 2012. Service users were aged 16+; however, the average age was 59 years old. The most common difficulties (based on self-report) were social isolation (35%), depression (32%), low mood (18%), loneliness (15%) and anxiety (10%). Notable findings include 70% of all service users who attended at least two assessments, demonstrated an increase in perceived wellbeing. Amongst service users who took up at least one activity, their wellbeing increased by 87%. Finally, one-third of service users who began the New Routes process went on to take up hobbies, with an average of two hobbies each. Their wellbeing increased by 96% [26].

Although the majority of studies focus on adults, there is a small number that look at the effectiveness of social prescribing on the mental health of young people. One such study examined students aged between 16 and 24 years old. They compared those who took part in team sports, informal fitness groups and those who exercised alone at least once a week. A six-month follow up found that taking part in group physical activity, whether in team sports or informally, was associated with better mental health outcomes and decreased depressive symptoms, compared to independent exercise. Students exercising in groups also reported feeling more connected to people around them [27]. Similarly, a cross-sectional study of students in Norway found that physical activity in a sports club was associated with significantly fewer depressive symptoms, compared to independent exercise [28]. This large-scale study consisted of over 17,000 secondary school students, and results were based on self-report measures. Doré et al. also carried out a cross-sectional study of 16–24-year-olds in Canada which echoed these results: individuals involved in team sports had lower self-reported anxiety and depression scores than those who engaged in physical activity individually [29].

Clarke et al. explored the effects of a variety of group activities on secondary school students in the UK. Activities included sports, music and drama-based interventions; significant improvements were found in students’ self-esteem, confidence and emotional regulation [30]. Additionally, a review of studies exploring the effects of group creative activities on the health and well-being of children, found that participation had a positive effect on self-confidence and self-esteem [31]. Both of these studies share the same limitation: they focus on activities that took place in community settings or were extra-curricular; young people did not necessarily have to have any diagnoses and no details are given regarding this or other variables, such as involvement with clinical mental health services. 

### 1.3. Mechanisms

In addition to evidencing the benefits of social prescribing on mental health, a number of studies have explored the possible mechanisms behind this. Hassan et al. focused on an NHS-run social prescribing service in Liverpool called Life Rooms. Service users are those from disadvantages backgrounds around Liverpool and Sefton who are experiencing mental health issues. The service does not have any formal requirements for access and individuals include Mersey Care service users, those under primary care or receiving support from public or third sector organisations, as well as the general public. No further details are provided regarding the types or severity of mental health issues experienced by service users. Individuals have a choice regarding the type of support they access, but this typically takes the form of learning opportunities or social support. The service includes pathways advice; this delivers social prescribing support through a one-to-one daily drop-in with a pathways advisor who helps service users to co-create their own social prescription. The authors reported that the data identified four mechanisms that seemed to be responsible for the success of the service: social belonging, resourcefulness and accessibility, social inclusion and connectedness and self-development and independence [15]. 

Furthermore, Kelly et al. reviewed the effectiveness of ‘Men’s Sheds’ on the mental health of adult males in Scotland [32]. Men’s Sheds are community-based organisations delivering practical and social activities that encourage positive health behaviours. Of the 62 Shed members interviewed, 40 reported having an existing illness or injury diagnosed by a health professional. The qualitative data from this study indicated that the service provided three specific inputs that directly impacted the ability of members to improve their health: Practical/educational, social/interactive and inclusive/supportive. Further findings included a decrease in excessive alcohol use in members with addiction issues. The members attributed this to having the opportunity to talk to health professionals, as well as other Shed members about sensitive and personal issues in a supportive environment. Members also reported that Sheds offer a socially acceptable ‘safe space’ for men to talk openly about their mental health concerns, where opportunities may have not have previously existed in typical male environments, such as bars and sports clubs.

There is a recognised connection between mental health and school connectedness in young people. Shochet and colleagues examined the relation between school connectedness (i.e., the extent to which students feel accepted, valued, respected and included in the school) and mental health symptoms in students ages 12 to 14. Measures were taken twice, 12 months apart; school connectedness correlated extensively with depression, anxiety and general functioning at both times points. School connectedness also predicted depressive symptoms one year later for both boys and girls, anxiety symptoms for girls and general functioning for boys [33]. These findings are supplemented by a longitudinal study of 2678 young people, which demonstrated that when connectedness in school is combined with social connectedness (such as groups outside of school), the risk of developing depressive symptoms is significantly reduced [34].

### 1.4. Linking Leeds

The evidence available for the use of social prescribing in young people is much scarcer than that for adults. However, based on the aforementioned statistics regarding the prevalence of mental health issues in young people, in addition to long waiting lists for treatment, it is important to explore and supplement the evidence base for this intervention. The objective of this paper is therefore to evaluate the impact of a current social prescribing service on the mental health and wellbeing of young people; specifically, a Leeds-based service named Linking Leeds.

Linking Leeds is an integrated, free social prescribing service for people in all areas of Leeds. It is made up of seven local partners, commissioned by NHS Leeds CCG and led by the Community Links charity.

Linking Leeds provides social prescribing for people aged 16 years and above; however, the current paper focuses on service users aged between 16 and 25. Their aim is to connect people to services and activities in their community in order to benefit overall health and mental wellbeing. Support provided includes housing and financial advice as well as support to engage in social and meaningful activities.

There are three ways to access Linking Leeds—GP introduction, self-referral or introduction from an alternative professional.

## 2. Materials and Methods

### 2.1. Participants

The data pertained to Linking Leeds service users aged between 16–25. These young people were referred to the service by their GP, a third-party professional or via self-referral and had been identified as struggling to cope with stress related to non-medical issues. These issues are predominately linked to social isolation, socioeconomic issues (such as finances, housing, or welfare needs), or have low-medium mental health issues that are creating health inequalities.

### 2.2. Materials

Data was collected using four different assessment tools, as detailed below.

#### 2.2.1. UCLA Loneliness Scale

This is a 20-item scale (see Appendix A) designed to measure one’s subjective feelings of loneliness as well as feelings of social isolation. The scale has been found to have good internal reliability (Cronbach’s α ranges from 0.89 to 0.94) and validity (statistically significant correlations with several other established measures of loneliness, including the NYU Loneliness Scale (*r* = 0.65) and the Differential Loneliness Scale (*r* = 0.72) [35]). Service users rate each item as either: O (“I often feel this way”), S (“I sometimes feel this way”), R (“I rarely feel this way”), or N (“I never feel this way”). 

Linking Leeds gather data for three of the twenty items, which cover three aspects of loneliness: Lacking companionship, feeling left out and feeling isolated from others. These three items are:

How often do you feel that you lack companionship?

How often do you feel left out?

How often do you feel isolated from others?

#### 2.2.2. Office for National Statistics (ONS) Personal Wellbeing Scale

Personal well-being (PWB) is part of the wider Measuring National Well-being (MNW) Programme at the Office for National Statistics, which aims to provide accepted and trusted measures of the nation’s well-being. It has been found to have good internal reliability (Cronbach’s α = 0.90) and validity (e.g., scores positively correlate with health confidence, *r* = 0.60) [36]. Their personal well-being assessment consists of four measures (often referred to as the ONS4, see Appendix B), which capture three types of well-being: evaluative, eudemonic and affective experience. 

#### 2.2.3. Short Warwick-Edinburgh Mental Wellbeing Scale (SWEMWBS)

The 7 items in the SWEMWBS were originally drawn from the full version of the WEMWBS. The original version of the WEMWBS contains 14 positively phrased items (see Appendix C) and was developed to enable the monitoring of mental wellbeing in the general population and the evaluation of projects, programs and policies which aim to improve mental wellbeing. Both scales are considered to be robust when applied in population, community, educational, occupational and clinical settings and studies have confirmed internal reliability (Cronbach’s α = 0.89) and validity (e.g., significant positive correlation between scores on the SWEMBS and the Satisfaction With Life Scale, *r* = 0.66) [37].

Each item is scored on a 5-point Likert-type scale ranging from ‘None of the time’ to ‘All of the time’. The raw score is calculated as the total across the seven items, none of which can be absent, and is then transformed via a conversion table into a metric score, which should be suitable for parametric analyses. Young people are asked to describe their experiences over the past two weeks. 

#### 2.2.4. Wellbeing Wheel

The Wellbeing Wheel is a measure created by the Linking Leeds service. It is adapted from the Wheel of Wellbeing, introduced by Sadigh and Sadigh in 2008 as part of the Big Lottery Well London’s programme [38]. The original is an ongoing collaboration between the Mental Health Promotion Team at South London and Maudsley NHS Foundation Trust, Uscreates and Implemental (formerly Maudsley International). The wheel of wellbeing focuses on six aspects of life: Body, mind, spirit, people, place and planet. Linking Leeds adapted this to measure aspects relevant to their service and service users:

Social Networks & Relationship 

Looking after yourself

Managing money

Housing

Meaningful use of time

Emotional Wellbeing 

Service users are presented with an image of the wheel (see Appendix D) and asked to indicate how well they feel they are managing each respective aspect.

### 2.3. Design

An outcome evaluation was conducted to assess changes over time in wellbeing outcomes targeted by the Linking Leeds program. This is therefore a non-experimental design that does not consist of a control group. An anonymised data set, in accordance with NHS ethics, was provided by Linking Leeds and no personal, identifiable information was used.

### 2.4. Procedure

This evaluation reports on data routinely collected by the Linking Leeds service, between 9 January 2019–11 January 2020. The data was collected in accordance with the ethic code of practice for Leeds and York Partnership Foundation NHS Trust. These dates are the equivalent of one year’s involvement in the service. On average, service users receive what equates to one contact with a wellbeing coordinator per week. A contact can be either face to face or via phone/video, dependent on individual needs.

In accordance with NHS ethics for service evaluation, Linking Leeds provided an anonymised data set. The ethical standards for Leeds and York Partnership Foundation Trust were adhered to throughout. No personal, identifiable information was used.

## 3. Results

### 3.1. UCLA Loneliness Scale

Service users completed the measure upon entry to the service and again upon exit. A summary of the differences between service users’ scores at the beginning and end can be found in Table 1. Data were available for 21 service users. Results show that 38.1% (*n* = 8) of participants’ lack of companionship scores decreased, 14.3% (*n* = 3) increased and 47.6% (*n* = 10) remained the same. Furthermore, 52.4% (*n =* 11) of participants reported feeling left out less on exit compared to entry of the service, while 14.3% (*n* = 3) of scores increased and 33.3% (*n* = 7) remained the same. In terms of isolation scores, 61.9% (*n* = 13) of these decreased, 9.5% (*n =* 2) increased and 28.6% *(n* = 6) remained the same. Finally, 71.4% (*n =* 15) of participants displayed decreased scores overall on the UCLA Loneliness Scale, while 19% (*n =* 4) of participant scores increased and 9.5% (*n =* 2) remained the same.

### 3.2. Office for National Statistics (ONS) Personal Wellbeing Scale

Service users completed the measure upon entry to the service and again upon exit. A summary of the differences between service users’ scores at the beginning and end can be found in Table 2. Data for this measure was available for 18 service users.

Results showed 55.5% (*n* = 10) of young peoples’ happiness scores increased, 22.2% (*n* = 4) decreased and 22.2% (*n =* 4) remained the same. Regarding anxiety, 50% (*n* = 9) of young peoples’ scores decreased, 44.4% (*n* = 8) increased and 5.6% (*n* = 1) remained the same. Additionally, 55.5% (*n =* 10) of life satisfaction scores increased, 27.8 (*n* = 5) decreased and 16.7% (*n* = 3) remained the same while 66.7% (*n* = 12) of service users reported an increase in feeling that things they do in life are worthwhile, 22.2% (*n* = 4) reported a decrease and 11.1% (*n* = 2) remained the same. Finally, 66.7% (*n* = 12) of scores on this scale increased overall, 27.8 (*n* = 5) decreased and 5.6% (*n* = 1) remained the same.

### 3.3. Short W3.3 Warwick-Edinburgh Mental Wellbeing Scale (SWEMWBS)

Service users completed the measure upon entry to the service and again upon exit. A summary of the differences between service users’ scores at the beginning and end can be found in Table 3. Data were available for 21 service users.

Results showed 52.4% (*n =* 11) of service users’ optimism about the future increased, 9.5% (*n =* 2) decreased and 38.1% (*n*= 8) remained the same, while 61.9% (*n =* 13) felt more useful after utilising the service compared to before, 14.3% (*n =* 3) felt less useful and 23.8% (*n =* 5) remained the same. Additionally, 71.4% (*n =* 15) of young people exhibited increased scores on the ‘feeling relaxed’ sub-scale, 9.5% (*n =* 2) of scores decreased and 19% (*n* = 4) remained the same.

Regarding ability to deal with problems, 42.9% (*n =* 9) of service users reported an increase on this scale, 9.5% (*n =* 2) reported a decrease and 47.6% (*n =* 10) remained the same. On the ‘thinking clearly’ scale, 47.6% (*n =* 10) of scores increased, 9.5% (*n =* 2) decreased and 42.9% (*n =* 9) remained the same. Furthermore, 61.9% (*n =* 13) of service users reported an increase in feeling close to other people, 4.8% (*n =* 1) reported a decrease and 33.3% (*n =* 7) of scores remained the same.

Finally, 57.1% of service users (*n =* 12) felt they could make up their own mind about things more often after engaging with the service, 14.3% (*n =* 3) reported a decrease on this item and 28.6% (*n =* 6) of scores remained the same, while 80.9% (*n =* 17) of service users’ scores increased overall, 14.3% (*n =* 3) decreased and 4.8% (*n =* 1) remained the same.

### 3.4. Wellbeing Wheel

Service users completed the measure upon entry to the service and again upon exit. A summary of the differences between service users’ scores at the beginning and end can be found in Table 4. Data were available for 14 service users. On this scale, an increase in score reflects a decrease in the service user’s perceived level of required support.

Results showed that on the social networks and relationship scale, 57.1% (*n =* 8) of service users’ scores increased, none decreased and 42.9 (*n =* 6) remained the same. Similarly, 57.1% (*n =* 8) of ‘looking after yourself’ scores increased, 7.1% (*n =* 1) decreased and 35.7% (*n =* 5) remained the same. Furthermore, 64.3% (*n =* 9) of service users reported requiring less support with managing money, none reported requiring more support and 35.7% *n =* 5) of scored remained the same.

After engaging with the service, 42.9% (*n =* 6) of service users required less support with housing, 14.3% (*n =* 2) reported requiring more support and 42.9% (*n =* 6) of scores remained the same. Increased scores were elicited for the majority of service users on the final two scales: 71.4% (*n =* 10) of meaningful use of time scores increased while 7.1% (*n =* 1) decreased and 21.4% (*n =* 3) remained the same; 85.7% (*n =* 12) of emotional wellbeing scores increased, 7.1% (*n =* 1) decreased and 7.1% (*n =* 1) remained the same. Finally, 100% (*n =* 14) of service users’ overall scores increased, reflecting a decrease in their support requirements.

## 4. Discussion

This evaluation supports the literature that there are two main mechanisms that underpin social prescribing in young people (25 years and under): social connectedness and behavioural activation. Social connectedness reduces loneliness and provides peer support which can reduce feelings of depression and anxiety [39]. Behavioural activation involves regularly engaging in activities that are positively reinforcing, which can improve independence skills and self-esteem [40]. 

### 4.1. Social Connectedness

The majority of young people’s loneliness scores decreased after engaging with the Linking Leeds service; 50% felt their social networks and relationships had improved and 71% reported a reduction in loneliness. This supports the research conducted by Thomas et al. who found the main benefit of social prescribing was the reduction of social isolation, as the young people were able to build a supportive social network [17]. Young people with poor mental health often become isolated, which can have a further negative impact on wellbeing [41]. 

There is an increasing recognition that there is a connection between mental health and social connectedness in young people; one study found poor school connectedness and predicted the development of depressive symptoms within 1 year [33] while another concluded that when school connectedness is combined with other social connections (such as groups outside of school), the risk of developing depressive symptoms is significantly reduced [34]. 

It is of note that 50% of the young people in this study did not describe an improvement in social relationships from the intervention. This may be due to them already having an established network of friends or because they did not expand their network through the intervention. Interestingly, a larger number described reduced loneliness which could indicate that the act of social interaction has an impact even when it does not result in a new relationship being formed.

### 4.2. Behavioural Activation

Behavioural activation is the act of regularly engaging in activities that are positively reinforcing for the individual [42]. This involves the young person engaging or re-engaging in activities or interests that are generally enjoyable. Behavioural activation has a robust evidence base in adulthood for people with depression [43,44] and there is now a growing evidence base in children and young people [40,45]. The current study found that over 70% of young people who engaged with Linking Leeds displayed increased scores regarding “Meaningful use of time”. Similarly, over 60% of “Feeling useful” scores increased and young people reported an increase in feeling that what they do is worthwhile. Torrissen and Stickley found similar conclusions; they report that engaging in a meaningful activity (in this case a theatrical group) led participants to feel empowered and more hopeful [46]. 

### 4.3. Impact of Social Prescribing on Mental Health

A systematic review of 86 social prescribing interventions for adults found increases in self-esteem, confidence and positive mood [18]. Another review examining social prescribing in schools concluded that nurturing groups have a significant positive impact on the emotional wellbeing of young people [47]. The results from the current study support the growing body of evidence that social prescribing can be an effective intervention for young people. Participants indicated their sense of happiness and life satisfaction increased after utilising the Linking Leeds service and they had a greater sense of optimism about the future. Furthermore, 81% of participants who completed the SWEMWS and 66% who completed the ONS personal wellbeing scale, felt their sense of wellbeing had improved. This increased to 100% of participants when looking at the Wellbeing Wheel score. 

Multiple systematic reviews [17,18,19,23] on social prescribing interventions in adults have found a positive impact on depression and anxiety symptoms. Affective disorders make up a significant proportion of all referrals to CAMHS; however, there is often a wait for appropriate therapeutic treatment. Linking Leeds found that after participation there was an improvement in anxiety scores for 44% of young people and 71% described themselves as “more relaxed”. Grant and colleagues found that a social prescribing intervention conducted with 161 young people resulted in significant improvements in anxiety scores [25], while Dore et al. found group exercise significantly improved mental health outcomes of 16–24-year-olds, compared to those who engaged in independent exercise [27]. 

One of the main impacts of social prescribing in young people appears to be an improvement in self-esteem. Clarke et al. explored the effects of sports, music and drama-based interventions on secondary school students in the UK. Participation in these interventions resulted in significant improvements in students’ self-esteem, confidence and emotional regulation [30]. Linking Leeds users felt “more useful” and confident at making decisions post intervention. Das et al. concluded creative social activities including music, dance, singing, drama and visual arts had a positive effect on self-esteem and self-confidence [48]. Garcia-Poole et al. conducted a large case-control study in young people engaging in a community social prescribing program. They found a significant positive impact on self-esteem compared to young people who were not engaged in the program [49]. 

### 4.4. Limitations

The Linking Leeds service is confined to the city of Leeds (which does include some rural areas). Leeds has a population of around 520,000 of which 85% are white, British ethnicity [50]. 20% of people under the age of 20 live in poverty compared to the national average of 17% [51]. For these reasons, this study should not be considered broadly generalizable to the UK population. Due to the nature of the study, there was no randomisation or control group and not all eligible young people took up the offer of the Linking Leeds intervention. This could have resulted in selection bias. The small sample size (14–21 participants) reduces any broader conclusions being drawn; however, it does fulfil the aims of this study.

## 5. Conclusions

The current difficulties young people have accessing services due to long waiting lists for CAMHS requires an urgent assessment of viable alternatives to formal therapeutic or medical interventions. There is an evidence base to demonstrate the efficacy of social prescribing in adult populations and a growing evidence base in young people. This study demonstrates the use of a social prescribing intervention used to help young people waiting for a formal therapeutic intervention from CAMHS. Social prescribing could be used while on a waiting list or as an adjunct to specialist therapeutic interventions. To date, there has been limited clinical or cost-effectiveness evidence; however, there are clear signals of efficacy from the research conducted. There is a need for randomised controlled trials in this area to determine the impact this intervention could have on young people’s mental health and its potential resource-saving effect. 

## Figures and Tables

**Table 1:** Differences in scores on the UCLA loneliness scale upon entry and exit to the service.

Lack of Companionship	Feeling Left Out	Isolation	Total Difference
0.00	0.00	−1.00	−1.00
−1.00	−2.00	−1.00	−4.00
−1.00	0.00	−1.00	−2.00
1.00	1.00	0.00	2.00
1.00	0.00	−1.00	0.00
−1.00	0.00	0.00	−1.00
0.00	1.00	0.00	1.00
0.00	2.00	2.00	4.00
0.00	−1.00	−1.00	−2.00
−1.00	−1.00	−1.00	−3.00
−1.00	−1.00	−1.00	−3.00
0.00	−2.00	−1.00	−3.00
0.00	−1.00	0.00	−1.00
0.00	−1.00	−2.00	−3.00
0.00	−1.00	0.00	−1.00
2.00	0.00	1.00	3.00
0.00	0.00	−1.00	−1.00
−1.00	−1.00	−1.00	−3.00
−2.00	−1.00	−2.00	−5.00
−2.00	−2.00	−1.00	−5.00
0.00	0.00	0.00	0.00

**Table 2:** Differences between scores on the Office for National Statistics Personal Wellbeing Scale upon entry and exit to the service.

Happiness	Reduction in Anxiety	Life Satisfaction	Feeling Things Are Worthwhile	Total Difference
3.00	−5.00	6.00	6.00	10.00
0.00	3.00	−1.00	0.00	2.00
5.00	5.00	6.00	8.00	24.00
−1.00	1.00	2.00	−1.00	1.00
1.00	5.00	0.00	1.00	7.00
2.00	4.00	1.00	3.00	10.00
1.00	−2.00	−1.00	5.00	3.00
−3.00	−3.00	−1.00	−10.00	−17.00
0.00	0.00	−2.00	1.00	−1.00
7.00	3.00	5.00	6.00	21.00
0.00	4.00	2.00	3.00	9.00
−2.00	−1.00	0.00	−3.00	−6.00
−4.00	−1.00	2.00	3.00	0.00
5.00	2.00	2.00	5.00	14.00
1.00	−2.00	4.00	4.00	7.00
0.00	−1.00	−1.00	−1.00	−3.00
3.00	−3.00	3.00	3.00	6.00
1.00	−3.00	0.00	0.00	−2.00

**Table 3:** Differences between scores on the Short Warwick-Edinburgh Mental Wellbeing Scale upon entry and exit to the service.

Optimism about the Future	Feeling Useful	Feeling Relaxed	Ability to Deal with Problems	Thinking Clearly	Feeling Close to Other People	Ability to Make Mind Up	Total Difference
1.00	1.00	2.00	2.00	1.00	0.00	1.00	6.44
−1.00	−1.00	1.00	−1.00	1.00	2.00	1.00	1.04
0.00	1.00	0.00	0.00	2.00	1.00	0.00	2.95
0.00	1.00	1.00	2.00	0.00	1.00	0.00	3.56
0.00	−1.00	1.00	0.00	−1.00	0.00	0.00	−0.99
0.00	0.00	0.00	0.00	0.00	0.00	0.00	0.00
0.00	0.00	1.00	0.00	0.00	0.00	−3.00	−1.10
0.00	0.00	1.00	0.00	0.00	0.00	1.00	1.27
0.00	0.00	0.00	0.00	0.00	1.00	1.00	1.62
0.00	1.00	2.00	3.00	0.00	2.00	1.00	5.70
1.00	1.00	1.00	3.00	1.00	2.00	1.00	6.85
1.00	2.00	2.00	2.00	2.00	1.00	1.00	7.75
1.00	1.00	2.00	0.00	0.00	2.00	1.00	8.32
1.00	1.00	−1.00	1.00	1.00	0.00	0.00	2.57
1.00	1.00	1.00	0.00	0.00	1.00	0.00	3.49
1.00	2.00	3.00	3.00	3.00	1.00	1.00	9.71
1.00	1.00	−1.00	2.00	1.00	−1.00	1.00	2.11
2.00	1.00	1.00	0.00	0.00	1.00	−2.00	2.14
4.00	2.00	2.00	2.00	4.00	4.00	2.00	17.11
1.00	0.00	0.00	0.00	−1.00	0.00	−1.00	−0.81
−1.00	−1.00	3.00	−2.00	1.00	1.00	1.00	1.62

**Table 4:** Differences between scores on the Wellbeing Wheel upon entry and exit to the service.

Social Networks & Relationships	Looking after Yourself	Managing Money	Housing	Meaningful Use of Time	Emotional Wellbeing	Total Difference
0.00	0.00	2.00	2.00	0.00	5.00	9.00
1.00	3.00	1.00	1.00	3.00	2.00	11.00
3.00	3.00	7.00	−1.00	4.00	3.00	19.00
0.00	0.00	0.00	0.00	0.00	5.00	5.00
0.00	6.00	0.00	−1.00	6.00	2.00	13.00
1.00	1.00	3.00	0.00	3.00	1.00	9.00
1.00	1.00	3.00	3.00	1.00	6.00	15.00
3.00	3.00	3.00	1.00	1.00	−1.00	10.00
0.00	2.00	0.00	0.00	6.00	4.00	12.00
1.00	1.00	1.00	1.00	3.00	7.00	14.00
3.00	0.00	0.00	0.00	2.00	0.00	5.00
0.00	0.00	0.00	0.00	0.00	3.00	3.00
1.00	−1.00	4.00	4.00	−4.00	1.00	5.00
0.00	0.00	4.00	0.00	4.00	2.00	10.00

## Data Availability

Not applicable.

## Appendix A

UCLA Loneliness scale.

## Appendix B

Office for National Statistics (ONS) Personal Wellbeing Scale.

## Appendix C

Short Warwick-Edinburgh Mental Wellbeing Scale (SWEMWBS).

Short Warwick Edinburgh Mental Wellbeing Scale (SWEMWBS) © NHS Health Scotland, University of Warwick and University of Edinburgh, 2008, all rights reserved.

## Appendix D

Wellbeing Wheel.
